# Resection and Rehabilitation for COVID-19 Associated Rhino-Maxillary Mucormycosis: A Case Report

**DOI:** 10.7759/cureus.39670

**Published:** 2023-05-29

**Authors:** Alladi Sneha, Santhosh Kumar M P, Murugesan Krishnan, Pradeep Dhasarathan, Hemavathy O R Muralidoss

**Affiliations:** 1 Oral and Maxillofacial Surgery, Saveetha Dental College and Hospitals, Chennai, IND

**Keywords:** rhino-maxillary mucormycosis, maxillofacial reconstruction, maxillofacial defect, quality of life, patient-specific implant

## Abstract

Often, patients with rhino-maxillary mucormycosis present with osteomyelitis and necrosis affecting the involved bone. Therefore, curative treatment involves a combination of antifungal therapy and surgical removal of the necrotic bone. In this case report, a 50-year-old female presented with pain in the right side of her face and was diagnosed with rhino-maxillary mucormycosis involving the right maxillary sinus, posterior maxilla, orbital floor, and zygomatic bone. To address the condition, a total maxillectomy of the right maxilla was performed. The post-surgical defect was packed using cotton leno-weave fabric, impregnated with soft paraffin and containing 0.5% chlorhexidine acetate dressing, which was changed every 3^rd^ day. After a six-month follow-up, satisfactory healing was observed. For, rehabilitation, a simple cast partial denture was used.

## Introduction

Mucormycosis is a fungal infection caused by Mucorales species. This species typically resides in the nasal and oral cavities of healthy individuals. Among the various forms of mucormycosis, rhino-maxillary mucormycosis is the predominant type, frequently found in uncontrolled diabetic patients with ketoacidosis. Additional contributing factors include iron overload, long-term glucocorticosteroid therapy, and neutropenia.

The pathogenesis of mucormycosis is an invasion of the fungus, causing endothelial damage leading to thrombosis and ischemia of the surrounding bone [1]. In the rhino-maxillary form, the infection enters the host through the nasal passage and extends through the paranasal sinuses, palate, orbit, and brain. A pathognomic feature is a reddish-black nasal turbinate and septum. Treatment of rhino-maxillary mucormycosis often involves surgical removal of the necrotic bone in combination with antifungal therapy.

During the COVID-19 pandemic in India, there was a surge in the incidence of mucormycosis disease [2]. Rhino-cerebral mucormycosis presents with osteomyelitis and necrosis of the affected bone. Therefore, curative treatment involves anti-fungal therapy and surgical removal of the necrotic bone. The maxillofacial defect created due to the surgical resection of the necrotic bone can significantly impact the patient’s quality of life, causing difficulty in speech, swallowing, mastication, and unesthetic appearance [3,4].

## Case presentation

**Figure 1:**
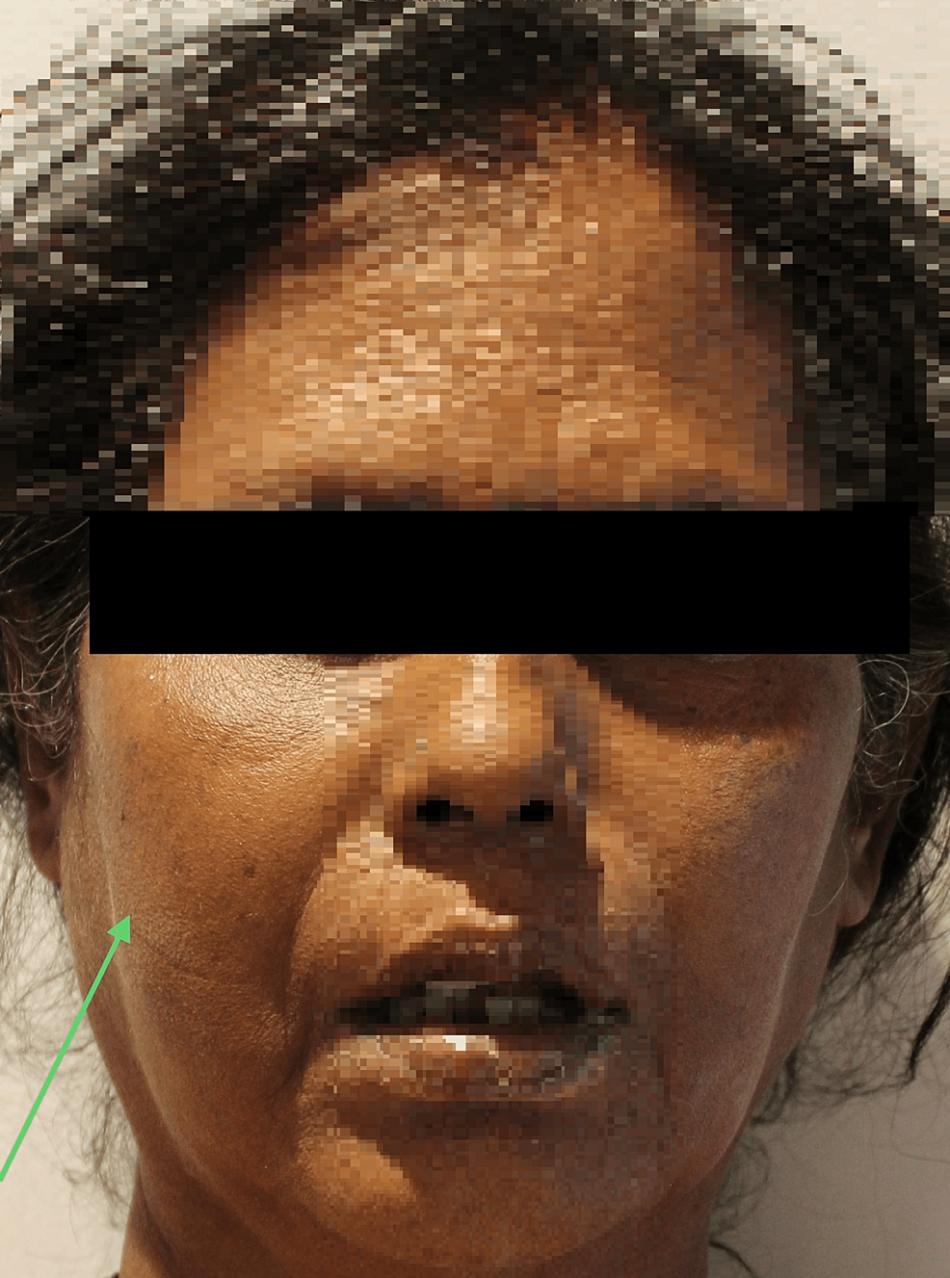
Pre-operative image This image depicts an extra-oral swelling observed on the right side of the face (arrow).

A 50-year-old female patient presented with a complaint of persistent right-sided facial pain for the past one month. The pain was intermittent and radiating in nature. The patient's medical history revealed a 10-year history of diabetes, for which she was receiving medication. The patient had visited another healthcare facility due to pus discharge in the right posterior maxillary region, leading to a provisional diagnosis of fungal osteomyelitis or maxillary sinus malignancy. The patient also gave a history of COVID-19 disease three months before the onset of pain in the right side of her face. On extra-oral examination, the swelling was noted on the right side of the face (Figure 1). The intra-oral examination did not reveal any visible pus discharge.

**Figure 2:**
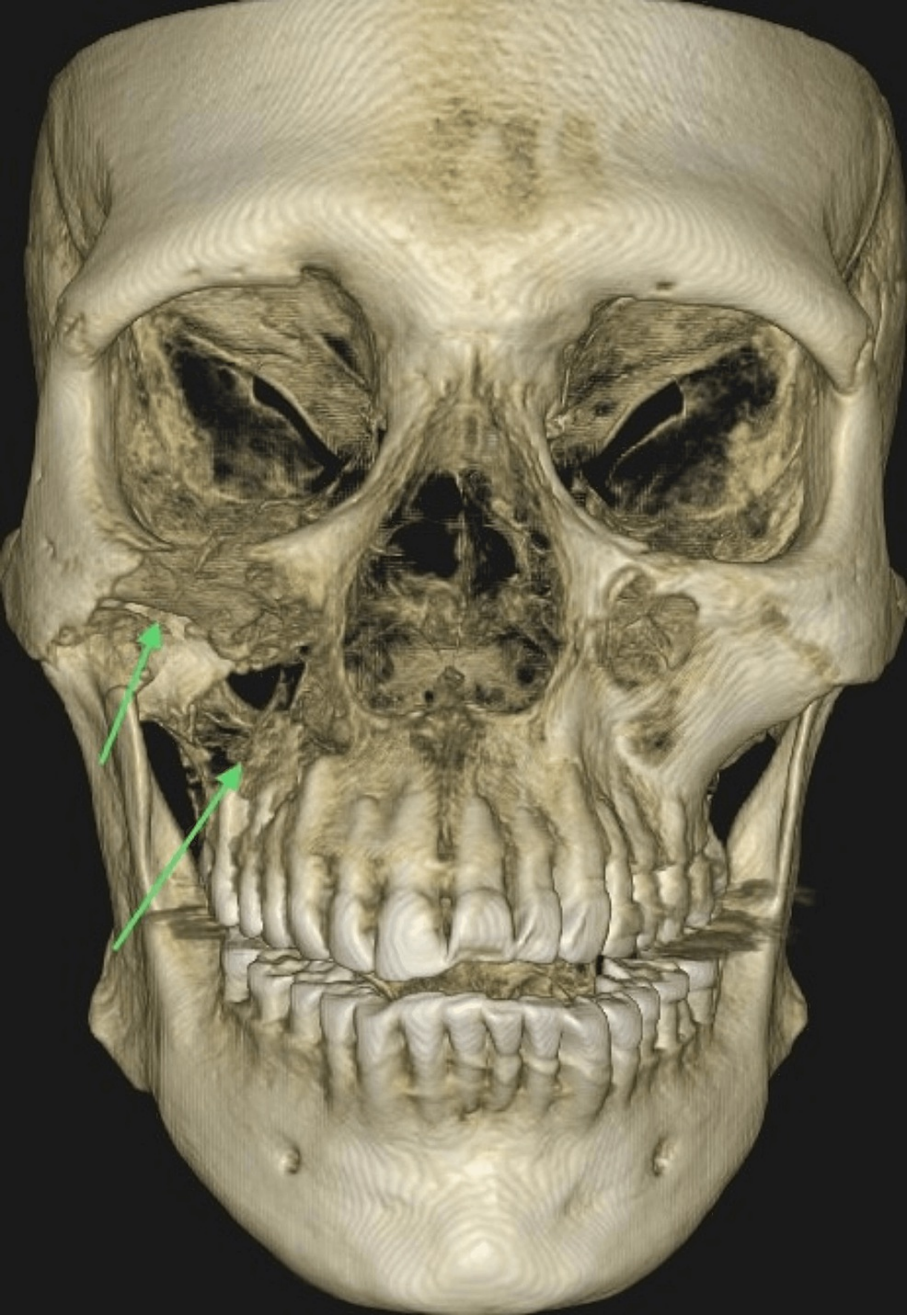
Pre-operative volumetric reconstruction of the CBCT findings CBCT: Cone Beam Computed Tomography This image depicts an osteolytic lesion in the right posterior maxilla, extending into the right orbital floor and zygomatic bone (arrow).

Radiographic evaluation showed radiopacity in the right maxillary sinus, with the obliteration of the medial and lateral sinus walls. Cone beam computed tomography (CBCT) revealed an osteolytic lesion in the right posterior maxilla, extending into the right orbital floor and zygomatic bone (Figure 2). The radiographic findings were consistent with osteomyelitis.

An incisional biopsy was performed in the posterior maxillary sinus, confirming the diagnosis of mucormycosis. The patient was initiated on antifungal therapy by administering oral posaconazole (200mg four times/day) for two weeks.

**Figure 3:**
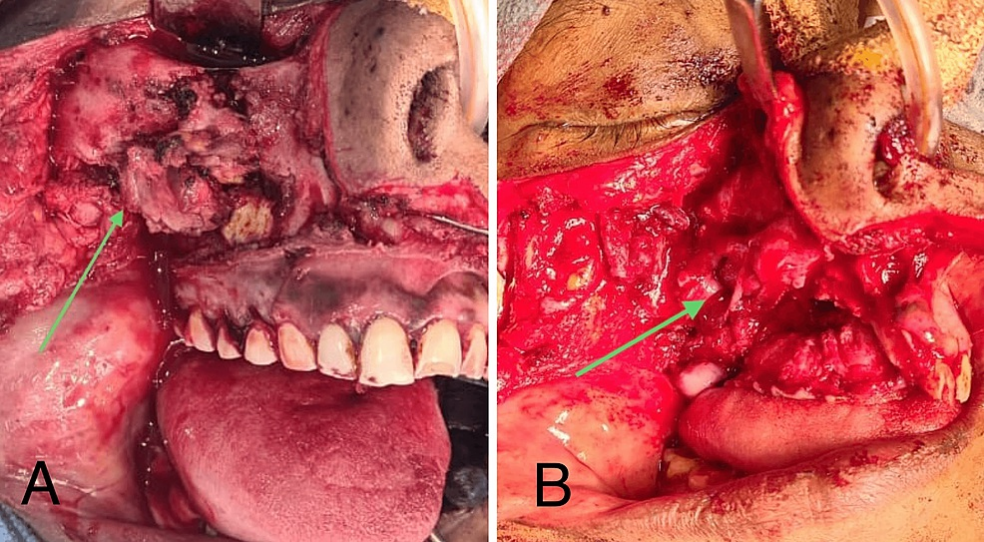
Intra-operative images (A) The exposure of the necrotic bone and (B) The surgical defect following the excision of the necrotic bone (arrow).

Surgical procedure: Under nasotracheal intubation, using standard sterilization protocols, a Weber-Ferguson incision was placed. Full-thickness mucoperiosteal flap elevation was performed, preserving the palatal mucosa. Total maxillectomy of the right maxilla, along with the infra-orbital margin, was performed, preserving the ligament of Lockwood (Figure 3). Curettage of the preserved palatal mucosa was done to eliminate residual fungal infection.

**Figure 4:**
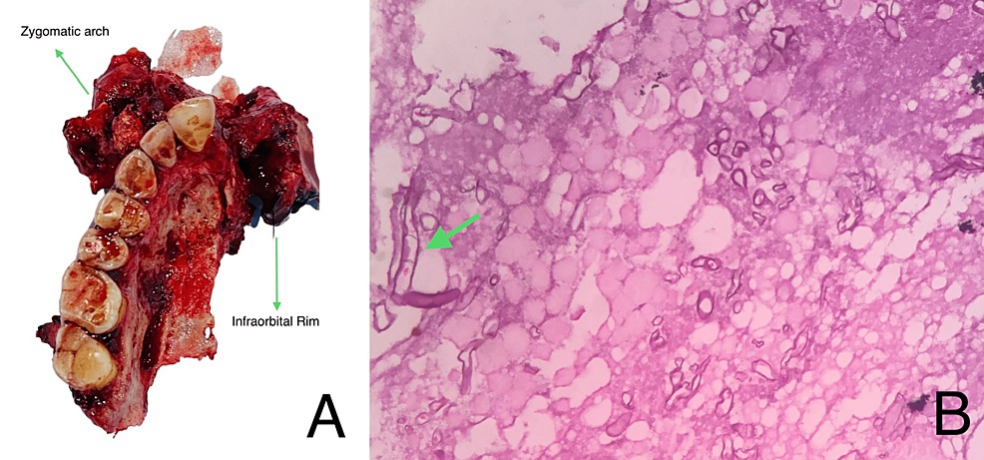
Histopathological diagnosis (A) The excised specimen concerning the zygomatic arch and infraorbital rim, and (B) the photomicrograph of the excised specimen showing numerous broad twisted aseptate fungal hyphae branching at an obtuse angle, along with an area of necrosis (10x, Hematoxylin and Eosin stain) (arrow).

Cotton leno-weave fabric impregnated with soft paraffin, containing 0.5% chlorhexidine acetate dressing, was packed into the defect. The closure was done in layers using 3-0 polyglactin and 4-0 nylon. A Ryle’s tube was placed postoperatively. The histopathological diagnosis of the excised specimen revealed mucormycosis (Figure 4). 

**Figure 5:**
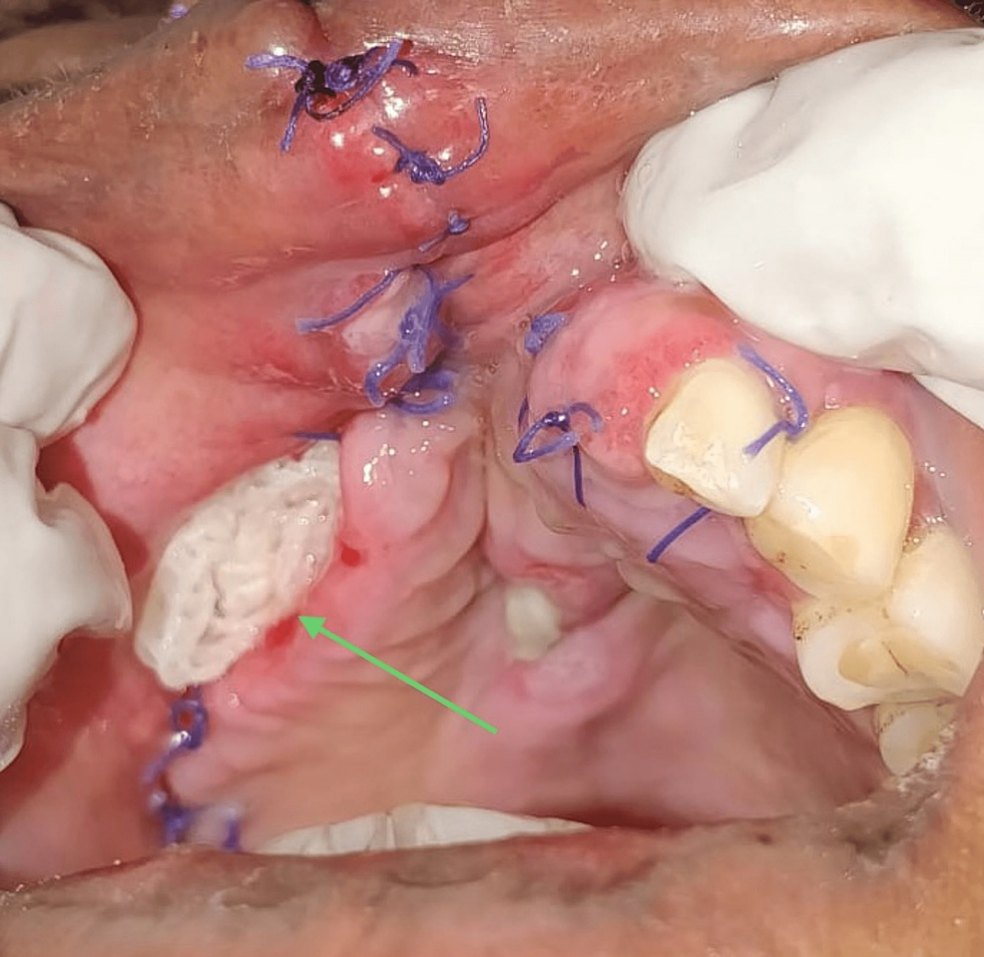
Post-operative healing This image depicts cotton leno-weave fabric, impregnated with soft paraffin and containing 0.5% chlorhexidine acetate dressing, packed into the defect for secondary healing on the 14^th ^postoperative day (arrow).

The cotton leno-weave fabric, impregnated with soft paraffin containing 0.5% chlorhexidine acetate pack was replaced every third day, which facilitated secondary wound healing with granulation tissue (Figure 5).

**Figure 6:**
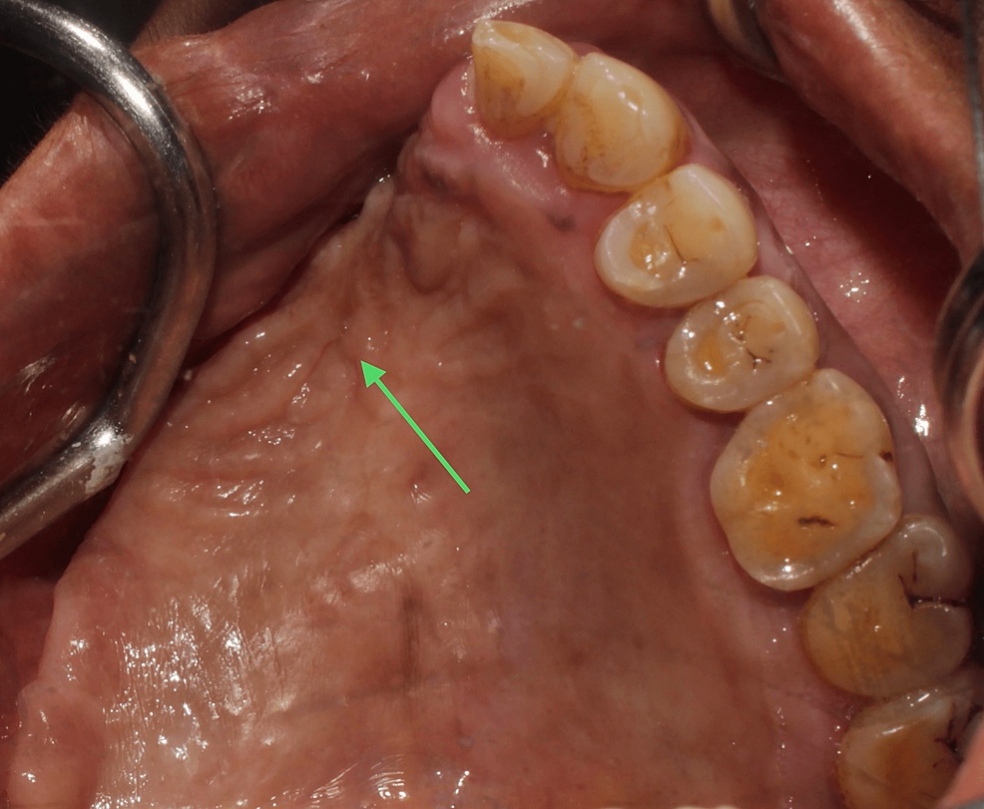
Postoperative intra-oral images This image depicts the post-operative intra-oral healing observed during a six-month follow-up (arrow).

Extraorally, the vertical level of the globe was maintained by preserving the ligament of Lockwood and the floor of the orbit. Postoperative healing was uneventful, with no signs of nasal regurgitation (Figure 6).

**Figure 7:**
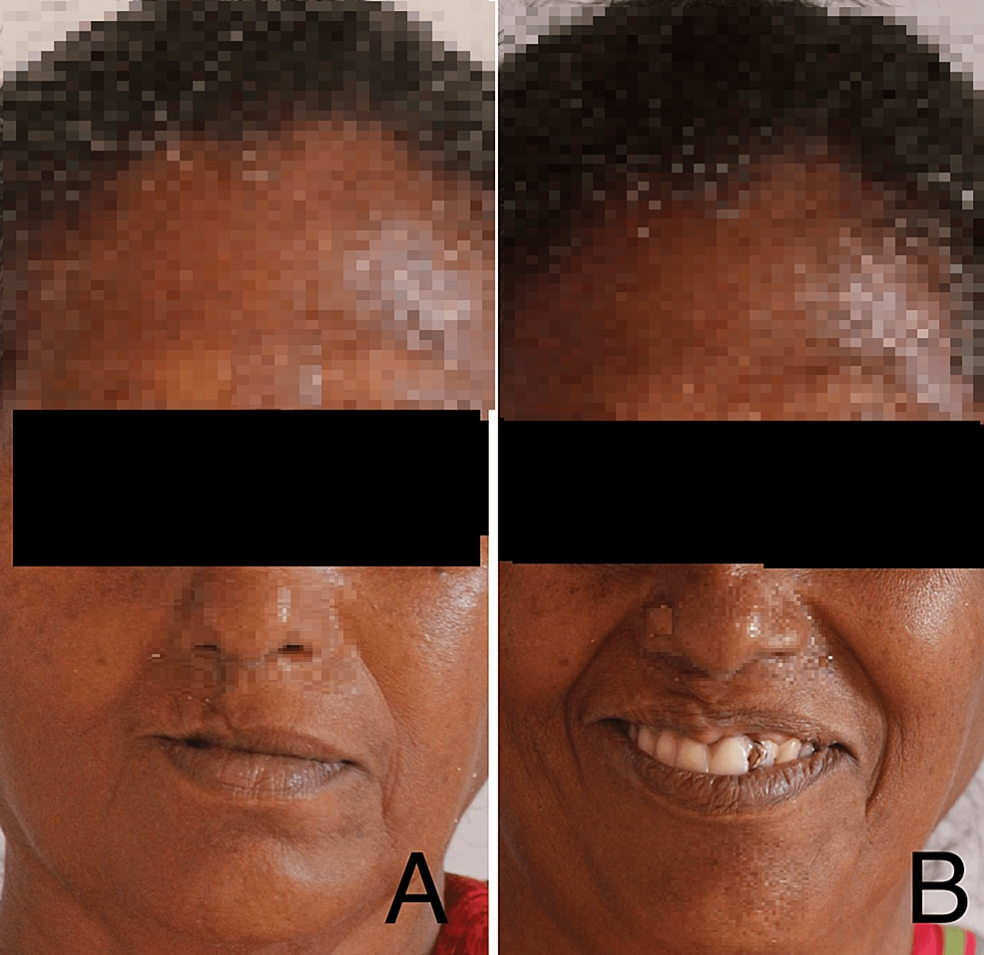
Six-month postoperative images This image depicts the post-operative picture taken during a six-month follow-up; (A) Post-operative extra-oral view (B) Smile with a cast partial denture.

**Figure 8:**
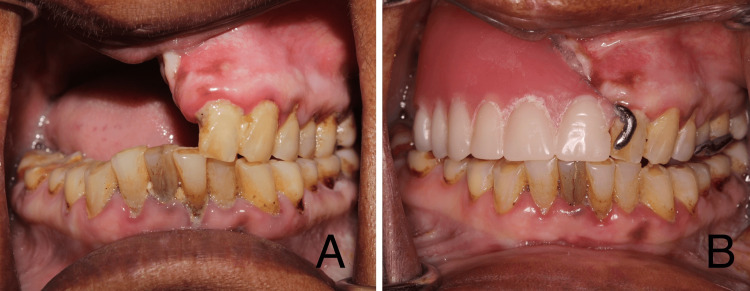
Postoperative and post-rehabilitation intra oral images This image depicts (A) The post-operative defect observed during a 6-month follow up (B) Rehabilitation using cast partial denture.

After uneventful healing, a six-month follow-up was conducted. Prosthetic rehabilitation was performed using a cast partial denture (Figures 7, 8).

**Figure 9:**
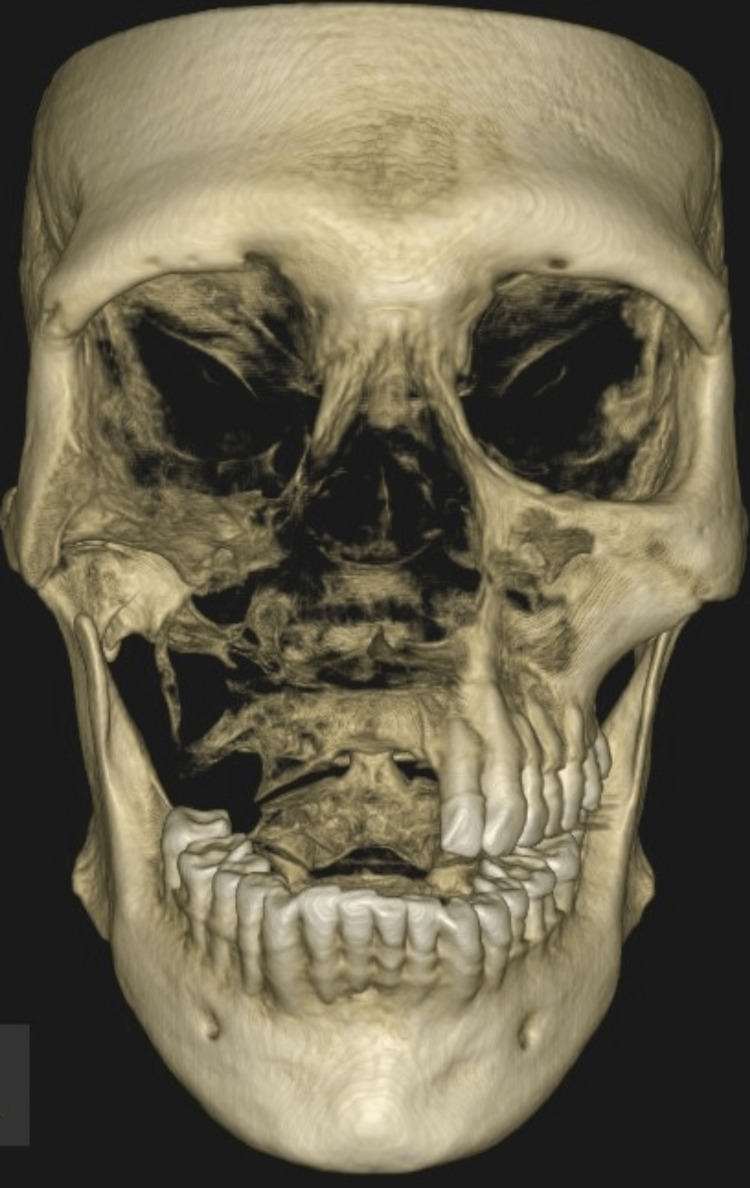
Postoperative volumetric reconstruction of the CBCT finding CBCT: Cone Beam Computed Tomography This image depicts a post-operative radiograph taken during a one-year follow-up.

A one-year follow-up radiograph was obtained to evaluate wound healing. The patient demonstrated uneventful healing both clinically and radiographically (Figure 9).

## Discussion

Mucormycosis is a fulminant disease, with the most common form being rhino-maxillary mucormycosis. This condition can leave patients mutilated and significantly decrease the quality of life [5]. Early diagnosis and prompt treatment are recommended due to the rapidly spreading nature of mucormycosis. Studies suggest patients with gingival swelling and histopathological features indicative of psedoepitheliomatous hyperplasia, should be considered a candidate for mucormycosis [6,7].

During the second wave of the COVID-19 pandemic in India, there was a surge in cases of rhino-maxillary mucormycosis, and the recommended treatment approach involved partial or total maxillectomy [8]. Preserving the palatal mucosa in patients undergoing maxillectomy for mucormycosis provides an opportunity for the defect to heal through the formation of secondary granulation tissue. The secondary granulation tissue is allowed to form using cotton leno-weave fabric impregnated with soft paraffin and containing 0.5% chlorhexidine acetate. The use of this dressing promotes the formation of a soft tissue seal between the oral, nasal, and antral cavities. Furthermore, in partially edentulous patients, preserving the palatal mucosa allows functional rehabilitation through the use of a simple cast partial denture.

Previous studies have shown the use of betadine-soaked gauze dressing to pack surgical defects [9]. However, in this particular case, we opted to use cotton leno-weave fabric impregnated with soft paraffin and containing 0.5% chlorhexidine acetate due to its antimicrobial properties against gram-positive and gram-negative bacteria. The healing of the oral mucosa showed satisfactory results. Further studies are required to evaluate secondary healing with antimicrobial packing.

Studies suggest impaired wound healing with the use of cautery, hence scalpel was used for incisions [10]. Though recent advances in suture material provide better wound healing, due to insufficient literature, closure was done using polyglactin suture [11,12].

In this particular instance, due to affordability, the patient was unwilling to undergo reconstruction of the defect using a free flap and patient-specific implants. Therefore, a cast partial denture was planned for the rehabilitation of the defect using an interdisciplinary approach. Though the most common type of management of reconstruction of defects is using free flaps and patient-specific implants, a cast partial denture can also be considered an alternative viable option [13].

## Conclusions

Mucormycosis is a disease that causes severe mutilation and impairment in a patient’s quality of life. In severely immunocompromised patients, such as uncontrolled diabetic patients, even minor dental procedures can stimulate fungal osteomyelitis. Early diagnosis and expeditious treatment are one of the major keys to success. While mucormycosis poses life-threatening risks, it often necessitates extensive resection. It is imperative to consider the rehabilitation of patients and the enhancement of their quality of life. Reconstruction and rehabilitation options for such patients should be decided considering the patient's psychological status, economic status, and individual needs, which may vary from case to case.
